# Editorial: Quantitative susceptibility mapping: technical advances and clinical applications

**DOI:** 10.3389/fnins.2023.1228061

**Published:** 2023-06-19

**Authors:** Hongjiang Wei, Xiaojun Guan, Peng Cao, Yuyao Zhang

**Affiliations:** ^1^School of Biomedical Engineering, Shanghai Jiao Tong University, Shanghai, China; ^2^Department of Radiology, The Second Affiliated Hospital, Zhejiang University, Hangzhou, China; ^3^Department of Diagnostic Radiology, Li Ka Shing Faculty of Medicine, University of Hong Kong, Hong Kong, Hong Kong SAR, China; ^4^School of Information and Science and Technology, ShanghaiTech University, Shanghai, China

**Keywords:** quantitative susceptibility mapping (QSM), MRI, brain, SWI, clinical applications

Quantitative susceptibility mapping (QSM) is a new magnetic resonance imaging (MRI) technique, which provides a non-invasive way to measure the spatial distribution of magnetic susceptibility (Liu et al., 2015a,b). QSM has been fueled by its unique ability to non-invasively quantify the spatial distribution of various biomolecules, an achievement that is promising to revolutionize our understanding of the brain's structure and function in health and disease (Li et al., 2023). This Research Topic, titled “*Quantitative Susceptibility Mapping: Technical Advances and Clinical Applications*,” shines a light on the rapid advances in this exciting field and the burgeoning array of clinical applications that these advances are making possible.

This Research Topic includes four original research papers and one review paper. A central theme that emerges from the papers in this Research Topic is the critical role that technical advances are playing in pushing the boundaries of QSM. Notably, the emergence of machine learning—and specifically deep-learning-based strategies—is propelling the field to new heights, offering the unprecedented potential for improved quantification accuracy and simplified preprocessing steps. He et al. proposed a novel k-QSM network to resolve dipole inversion issues in QSM reconstruction. The k-QSM network converts the results from truncated k-space division (TKD) into the Fourier domain as inputs. After passing through several convolutional and residual blocks, the ill-posed signals of TKD are corrected. Fuchs and Shmueli applied the incomplete spectrum approach to the field-to-source inverse problem encountered in quantitative magnetic susceptibility mapping (QSM). They showed that the incomplete spectrum QSM provides a new approach to handle the “ill-posed” regions in the frequency-space data input to QSM.

These sophisticated techniques may well prove pivotal in meeting some of the most significant challenges currently faced by QSM. However, the extraordinary potential of QSM extends far beyond these technical advances. Clinical applications of this technique are rapidly expanding, from tracking the progression of neurodegenerative diseases like Parkinson's disease and Alzheimer's disease (AD) to refining the implementation of deep brain stimulation surgery. Uchida et al. reviewed QSM as an imaging biomarker for Alzheimer's disease. They focused on the relationships among QSM, established biomarkers, and cognitive performance in AD. They also discussed the role of QSM as an imaging biomarker as well as the expectations and limitations of clinically useful diagnostic and therapeutic implications for AD. The application of susceptibility imaging has also been tested on 0.5T MRI by Qiu et al. to compare with the Susceptibility Weighted Imaging (SWI) at 0.5T and 1.5T. They found minor differences regarding the blood veins quantification. QSM has also been applied to investigate the brain using an ultrashort echo time sequence with a 3D cones trajectory (Jang et al.). They concluded that ultrashort echo time quantitative susceptibility mapping enables direct estimation of the magnetic susceptibility in the brain with a dramatically reduced total scan time with the use of a stretched 3D cones trajectory.

We hope that you will find the articles in this Research Topic both insightful and inspiring, and that they will spark new ideas and spur further research in this fascinating field. It is our privilege to present to you “*Quantitative Susceptibility Mapping: Technical Advances and Clinical Applications*”. The completion of this Research Topic involved the contribution of effort by authors and we want to take this opportunity to thank them all.

## Author contributions

HW, PC, XG, and YZ wrote the manuscript. All authors contributed to the article and approved the submitted version.
